# Systematic profiling of SARS-CoV-2-specific IgG responses elicited by an inactivated virus vaccine identifies peptides and proteins for predicting vaccination efficacy

**DOI:** 10.1038/s41421-021-00309-7

**Published:** 2021-08-17

**Authors:** Ming-Liang Ma, Da-Wei Shi, Yang Li, Wei Hong, Dan-Yun Lai, Jun-Biao Xue, He-Wei Jiang, Hai-Nan Zhang, Huan Qi, Qing-Feng Meng, Shu-Juan Guo, De-Ju Xia, Jin-Jun Hu, Shuo Liu, He-Yang Li, Jie Zhou, Wei Wang, Xiao Yang, Xiong-Lin Fan, Qing Lei, Wei-Jun Chen, Ce-Sheng Li, Xiao-Ming Yang, Si-Hong Xu, Hong-Ping Wei, Sheng-Ce Tao

**Affiliations:** 1 Key Laboratory of Systems Biomedicine (Ministry of Education), Shanghai Center for Systems Biomedicine, Shanghai Jiao Tong University, Shanghai, China; 2 National Institutes for Food and Drug Control, Beijing, China; 3 CAS Key Laboratory of Special Pathogens and Biosafety, Centre for Biosafety Mega-Science, Wuhan Institute of Virology, Chinese Academy of Sciences, Wuhan, Hubei China; 4 University of Chinese Academy of Sciences, Beijing, China; 5 Division of HIV/AIDS and Sex-transmitted Virus Vaccines, Institute for Biological Product Control, National Institutes for Food and Drug Control (NIFDC), Beijing, China; 6 Foshan Fourth People’s Hospital, Foshan, Guangdong China; 7 Key Laboratory of RNA Biology, Institute of Biophysics, Chinese Academy of Sciences, Beijing, China; 8 Department of Pathogen Biology, School of Basic Medicine, Tongji Medical College, Huazhong University of Science and Technology, Wuhan, Hubei China; 9 BGI PathoGenesis Pharmaceutical Technology, BGI-Shenzhen, Shenzhen, Guangdong China; 10 BGI Education Center, University of Chinese Academy of Sciences, Shenzhen, Guangdong China; 11 Sinopharm Wuhan Plasma-derived Biotherapies Co., Ltd., Wuhan, Hubei China; 12 China National Biotech Group Company Limited, National Engineering Technology Research Center for Combined Vaccines, Wuhan, Hubei China

**Keywords:** Immunology, Proteomic analysis

## Abstract

One of the best ways to control COVID-19 is vaccination. Among the various SARS-CoV-2 vaccines, inactivated virus vaccines have been widely applied in China and many other countries. To understand the underlying protective mechanism of these vaccines, it is necessary to systematically analyze the humoral responses that are triggered. By utilizing a SARS-CoV-2 microarray with 21 proteins and 197 peptides that fully cover the spike protein, antibody response profiles of 59 serum samples collected from 32 volunteers immunized with the inactivated virus vaccine BBIBP-CorV were generated. For this set of samples, the microarray results correlated with the neutralization titers of the authentic virus, and two peptides (S1-5 and S2-22) were identified as potential biomarkers for assessing the effectiveness of vaccination. Moreover, by comparing immunized volunteers to convalescent and hospitalized COVID-19 patients, the N protein, NSP7, and S2-78 were identified as potential biomarkers for differentiating COVID-19 patients from individuals vaccinated with the inactivated SARS-CoV-2 vaccine. The comprehensive profile of humoral responses against the inactivated SARS-CoV-2 vaccine will facilitate a deeper understanding of the vaccine and provide potential biomarkers for inactivated virus vaccine-related applications.

## Introduction

The coronavirus disease 2019 (COVID-19) is caused by severe acute respiratory syndrome coronavirus 2 (SARS-CoV-2)^1,2^. As of July 14, 2021, there has been 187 million cases of COVID-19 diagnosed, with 4.0 million deaths (https://coronavirus.jhu.edu/map.html)^3^. The genome of SARS-CoV-2 encodes four major structural proteins (spike (S), envelope (E), membrane (M), and nucleocapsid (N)), 15 nonstructural proteins (NSP1–10 and NSP12–16), and 8 accessory proteins^4^. Among them, the S protein, consisting of an N-terminal S1 fragment and a C-terminal S2 fragment, plays essential roles in viral attachment, fusion, and entry into target cells^5–9^.

Globally, the best and perhaps only way to return to normal life is to reach herd immunity through vaccination. The current efforts involve the fastest development of vaccines for an infectious disease in history^10,11^. According to the COVID-19 vaccine tracker (https://covid19.trackvaccines.org), 20 vaccines were approved for emergency use as of July 14, 2021, and 131 were in clinical trials. These vaccines can be classified into several groups, namely, RNA/DNA vaccines^12,13^, subunit vaccines^14,15^, and inactivated virus vaccines^16–18^, among others, with inactivated virus vaccines thought to be one of the most promising choices due to their potentially high efficacy, high safety, low cost, and high feasibility. Among the 16 SARS-CoV-2 inactivated virus vaccines currently in clinical trials, 10 are at clinical stage Phase III, including CoronaVac^17^, an inactivated virus vaccine from the Wuhan Institute of Biological Products^18^, and BBIBP-CorV^16,19^. BBIBP-CorV has already been approved in China and other countries, and demonstrates good protection efficacy^20^. These inactivated virus vaccines can trigger profound antibody responses in a variety of animal models, including nonhuman primates (NHPs), and humans. However, only weak induction of T_H_1 or T_H_2 cell responses has been observed in NHPs and humans^10^. In general, stimulation of an effective antibody response is the hallmark of a good inactivated vaccine and possibly the major mechanism underlying the effectiveness of inactivated SARS-CoV-2 vaccines^17^.

Theoretically, inactivated virus vaccines retain all of the antigenic components of the corresponding live virus, and it is important to understand the antibody responses of inactivated SARS-CoV-2 vaccines at the systemic level. Indeed, several key questions about the humoral immunity elicited by inactivated virus vaccines can be addressed through systematic analysis. For example, one can determine which SARS-CoV-2 protein or S protein peptide can induce significant antibody response, whether the antibody response against the inactivated virus vaccine differs from that of the live virus infection, the effectiveness of vaccination by comparing the antibody responses of vaccinated people and COVID-19 patients, and whether it is possible to identify peptide and/or protein combinations that may serve as surrogate biomarkers for convenient evaluation of the efficacy of vaccination and for differentiating COVID-19 patients from vaccinated individuals.

Additionally, a reliable, simple and cost-effective assay is needed to estimate the efficacy of protection against SARS-CoV-2 infection after immunization. Although the most reliable test is the neutralization assay of the authentic virus, this is impossible in practice because of the requirement of a level 3 biosafety facility. Even for neutralization assays of pseudoviruses, the requirements of sophisticated experimental skills and high cost limit their application. Other approaches include the sVNT (surrogate virus neutralization test) assay, which assesses IgGs against the S protein or its receptor binding domain (RBD)^21^. sVNT is promising but requires active RBD and human angiotensin-converting enzyme 2 (hACE2) proteins, which are relatively difficult to prepare than peptides.

Previously, we constructed a SARS-CoV-2 protein microarray and a peptide microarray with full coverage of the S protein, and we established a pipeline and analyzed >3000 COVID-19 serum samples with these two microarrays. Based on these microarrays, we constructed the SARS-CoV-2-specific antibody landscape at both the protein and peptide levels^22–25^.

To understand the IgG and IgM responses triggered by inactivated SARS-CoV-2 vaccine at the systemic level while taking advantage of the SARS-CoV-2 protein microarray and the spike protein peptide microarray, we in this study analyzed 59 serum samples from 32 healthy people immunized with the SARS-CoV-2 inactivated virus vaccine BBIBP-CorV^19^. We detected the profile of antibody responses at both the protein and peptide levels^25^. This profile was similar to those of convalescent patients and COVID-19 patients, though significant differences were also observed. Potential peptide/protein combinations capable of predicting the effectiveness of vaccination and differentiating vaccinated individuals from COVID-19 patients were also identified.

## Results

### SARS-CoV-2-specific antibody responses at the protein level are generally consistent between vaccinated volunteers and convalescent patients

**Fig. 1:**
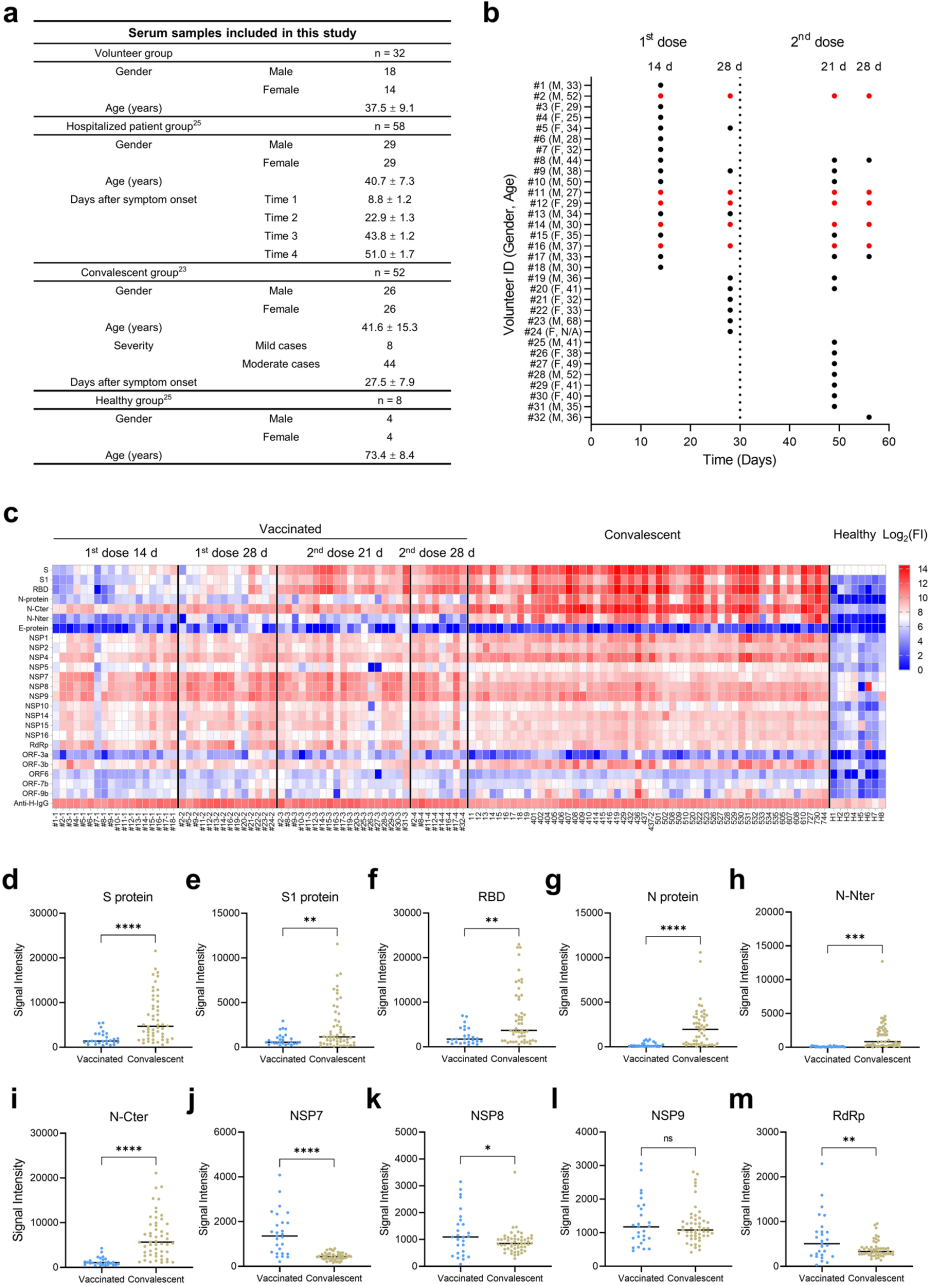
SARS-CoV-2-specific IgG responses after immunization with the inactivated virus vaccine. **a** Detailed information for the serum samples. **b** Sample collection after vaccination. Red dots represent volunteers who have four data points. **c** IgG profiles of vaccinated and convalescent sera against the SARS-CoV-2 proteome. FI, fluorescence intensity, was shown in log scale, i.e., log_2_(FI) ranges from 0 to 14. **d**–**m** IgG responses to selected proteins. Antibody responses against SARS-CoV-2 proteins, the S protein (**d**), S1 subunit (**e**), RBD (**f**), N protein (**g**), N-Nter (**h**), N-Cter (**i**), NSP7 (**j**), NSP8 (**k**), NSP9 (**l**), and RdRp (**m**). Sera were collected from vaccinated volunteers at 21 and 28 days after the 2nd vaccine dose (*n* = 27) and from convalescent patients (*n* = 52). The *P* value was calculated by the two-sided Student’s *t*-test. ^*^P < 0.05, ^**^P < 0.01, ^***^P < 0.005, ^****^P < 0.001, ns represents not significant.

To identify the SARS-CoV-2-specific antibody responses of individuals immunized with the inactivated virus vaccine, a cohort of 32 volunteers immunized with two doses of the inactivated vaccine BBIBP-CorV^16,19^ was included in this study (Fig. 1a). Sera were collected at four time points, i.e., 14 and 28 days after the 1st dose and 21 and 28 days after the 2nd dose, to investigate dynamic changes in SARS-CoV-2-specific antibody responses (Fig. 1b). In addition, a cohort of 52 convalescents^23^ and 58 hospitalized patients^25,26^ was included for comparison of SARS-CoV-2-specific antibody responses between vaccinated individuals and COVID-19 patients (Fig. 1a).

To obtain the global profile of SARS-CoV-2-specific antibody responses of vaccinated individuals, we analyzed serum samples from the volunteer group and the convalescent group utilizing the SARS-CoV-2 protein microarray that contained 3 structural proteins, 5 accessory proteins, and 12 nonstructural proteins^22^, and the data for the groups are presented together in Fig. 1c to obtain an overview of the IgG responses. The overall profiles of IgG responses at the 3rd and 4th time points were similar to those of convalescent patients, especially for S protein-related fragments, i.e., S, S1, and RBD. The 2nd dose of the inactivated virus vaccine is necessary to ensure a high level of neutralizing antibodies^16,18^, which was confirmed if we consider the IgG responses of S protein-related proteins as an index of neutralizing antibodies^21,27,28^.

For a more detailed analysis, we compared signals of the proteins individually after the 2nd dose to those of convalescent sera and found that S, S1, RBD, N, N-Nter (N-terminus of the N protein), and N-Cter (C-terminus of the N protein) signals were significantly lower (3.33-, 2.65-, 2.81-, 9.12-, 19.42-, and 5.16-fold, respectively) in the volunteer group than in the convalescent group (Fig. 1d–i). These results are consistent with the neutralization titer being lower in vaccinated volunteers than in convalescents^29^. Surprisingly, the NSP7 signal was consistently high at all four time points and was significantly higher (3.32-fold) in the volunteer group than in the convalescent group. Furthermore, the signals of the RNA-dependent RNA polymerase (RdRp) and NSP8 were 1.63- and 1.36-fold higher in the volunteer group, respectively, but NSP9 signals did not differ between the two groups (Fig. 1j–m).

To increase the comparability of longitudinal sera collected from vaccinated volunteers and COVID-19 patients, patient sera collected at four different time points after symptom onset were also analyzed. After infection of SARS-CoV-2, 3–7 days are typically required for symptoms to develop^30^. Thus, we set the four time points as 7–11 days (Time 1), 21–25 days (Time 2), 42–46 days (Time 3), and 49–53 days (Time 4) to match those of sera collected from vaccinated volunteers, i.e., 14 and 28 days after the 1st dose and 21 and 28 days after the 2nd dose, respectively (Fig. 1a). Because it is currently difficult to obtain COVID-19 sera, the protein microarray data generated in previous studies^25,26^ were examined instead. We compared overall IgG responses among the four time-matched sample sets (Supplementary Fig. S1), and the results showed consistent IgG profiles between the vaccinated volunteers and COVID-19 patients for Time 3 and Time 4. However, stronger IgG responses were observed for COVID-19 patients at Time 2 but not for the matched vaccinated volunteers at 28 days after the 1st dose. Specifically, after Time 1, IgG responses to S1, N, N-Nter, and N-Cter were significantly lower in vaccinated volunteers than in COVID-19 patients, whereas a reverse trend was observed for NSP7 at Time 3; no difference for NSP8, NSP9, and RdRp was detected (Supplementary Fig. S2). These results indicate that the IgG responses of vaccinated volunteers are generally weaker than those of COVID-19 patients.

### IgG responses to S, S1, and RBD of vaccinated volunteers correlate negatively with age

**Fig. 2:**
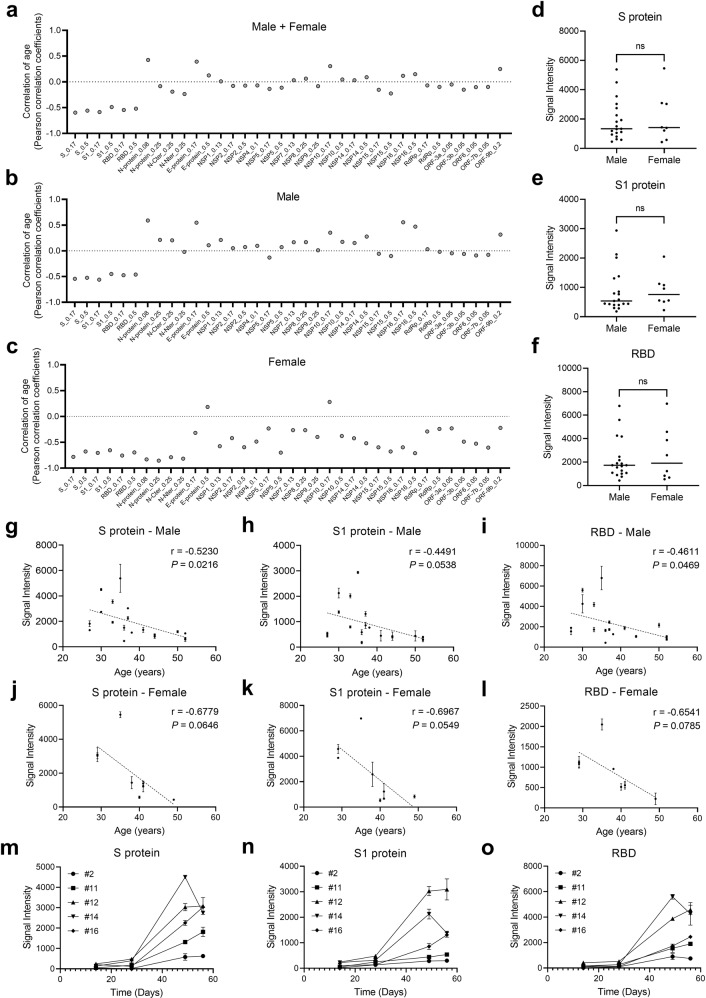
Correlation of protein (S, S1, or RBD)-specific IgG responses to age or gender. **a** Correlations of the overall IgG responses to age. **b**, **c** Correlations of IgG responses to age for males (**b**) and females (**c**). **d**–**f** IgG responses of males vs females to the S protein (**d**), S1 subunit (**e**), and RBD (**f**). The *P* value were calculated by the Student’s *t-*test. **g**–**l** Correlations of IgG responses to age for males and females for individual proteins, i.e., the S protein (**g**) and (**j**), S1 subunit (**h**) and (**k**), and RBD (**i**) and (**l**). **m**–**o** Trends of IgG responses to the S protein (**m**), S1 subunit (**n**), and RBD (**o**). Values are presented as the mean ± SEM. Unless otherwise stated, sera collected after the 2nd vaccine dose were analyzed.

It is known that older people usually have weaker immunity; therefore, a lower IgG response is anticipated after vaccination. In addition, the IgG response is proportional to age and differs between males and females among COVID-19 patients^22,28,31^. To evaluate whether IgG responses of vaccinated volunteers correlate with age at the protein level, we performed Pearson correlation analysis for all proteins vs age. Negative correlations were observed for S-related proteins in males and for S- and N-related proteins in females (Fig. 2a–c). In contrast, there were no significant differences between males and females regarding S-related proteins, i.e., S, S1, and RBD (Fig. 2d–f) or other proteins (Supplementary Fig. S3a–e). According to a detailed analysis, the correlation coefficient (r index) between the S, S1, and RBD signal intensity and age was ~–0.5 for males (Fig. 2g–i) and ~–0.7 for females (Fig. 2j–l). Although N correlated highly in females (Supplementary Fig. S3k), NSP7, NSP8, NSP9, and RdRp did not correlate or correlated only very weakly (Supplementary Fig. S3f–j, l–o). To determine dynamic changes in SARS-CoV-2-specific IgG responses, the microarray results of sera collected at 4 time points were plotted for 5 volunteers; except for the 4th time point of volunteer #14, the trends of IgG increased over time, especially after the 2nd vaccine dose, for S, S1, and RBD (Fig. 2m–o). Similar patterns were observed for the N protein (Supplementary Fig. S3p), with the trends for NSP7, NSP8, NSP9, and RdRp differing (Supplementary Fig. S3q–t). These results suggest that 2 doses of the inactivated virus vaccine are necessary and that IgG responses to S-related proteins correlate negatively with age. It is interesting to further explore whether the antibody responses to proteins are gender or age dependent. Thus, we performed stratification analysis with age and gender. Our results clearly demonstrated that, on protein level, there is no statistical difference between males and females, even when we stratify the samples in two age groups (Supplementary Fig. S4a–h).

### SARS-CoV-2-specific antibody responses at the peptide level of vaccinated volunteers are significantly weaker than those of COVID-19 patients

**Fig. 3:**
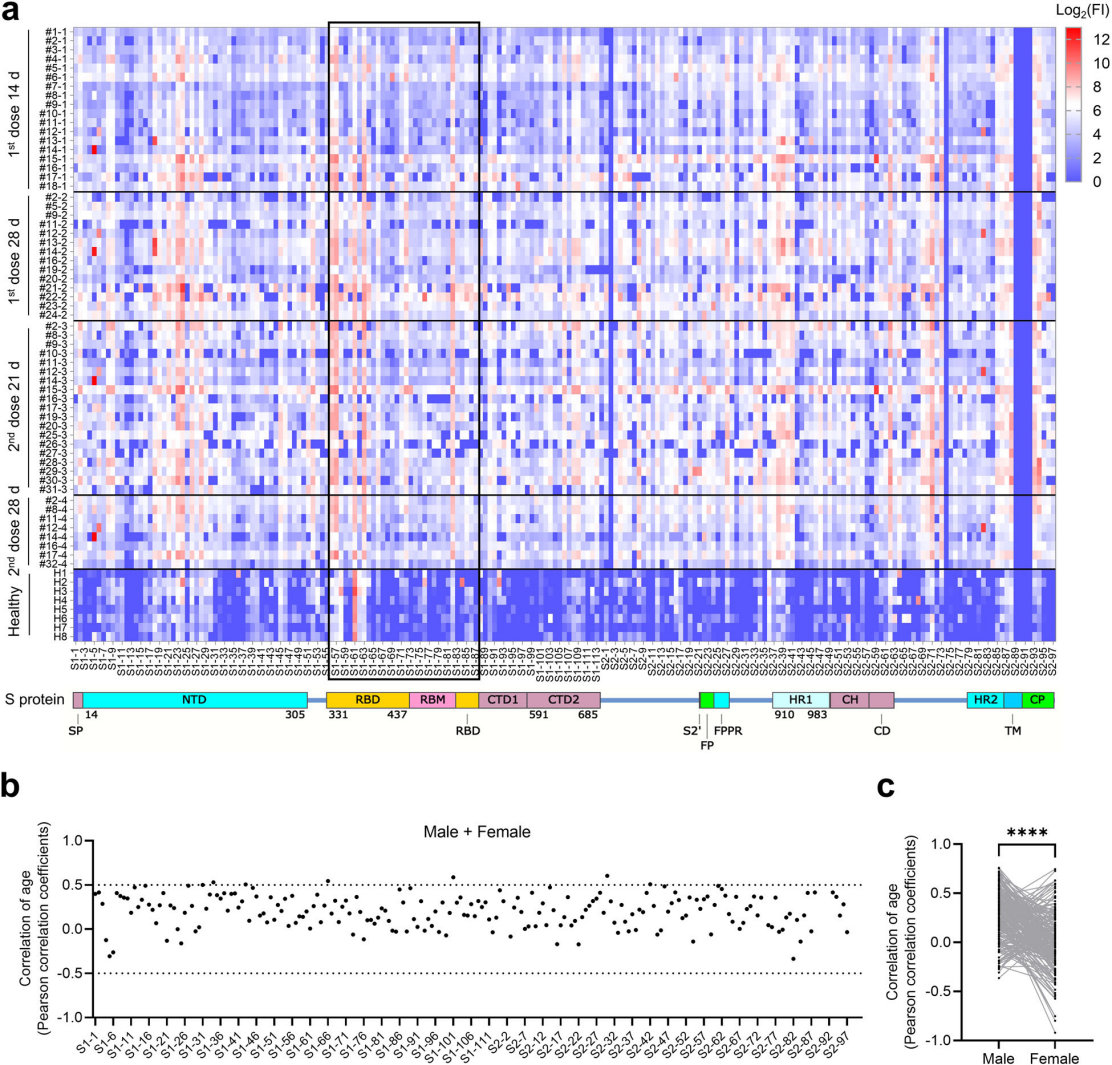
IgG responses to S protein peptides in vaccinated volunteers. **a** IgG responses of vaccinated individuals against S protein peptides. FI, fluorescence intensity, was shown in log scale, i.e., log_2_(FI) ranges from 0 to 12. **b** Correlation coefficient between the peptide-specific IgG response and age. **c** Correlation coefficients for male and female. Each line represents one peptide.

To obtain the global profile of the SARS-CoV-2 S protein-specific antibody responses of vaccinated individuals, we analyzed sera from the volunteer and convalescent groups using a peptide microarray containing 197 peptides across the S protein, and the length of the peptide is 12 amino acids, with 6 amino acids overlap for every two adjacent peptides^23,24^. Data for the volunteer group are presented together in Fig. 3a to obtain an overview of IgG responses. Several relatively hot regions were readily identified, e.g., S1-19–S1-28 (aa 109–174) of the N-terminal domain (NTD), S1-56–S1-63 (aa 331–384) of the RBD, S2-37–S2-41 (aa 902–937) of the heptad repeat 1 (HR1), and S2-85–S2-88 (aa 1190–1219) of the heptad repeat 2 (HR2). To evaluate whether IgG responses correlate with age at the peptide level, we performed Pearson correlation analysis for all S protein peptides vs age, though only a few peptides showed correlation values higher than 0.5 (Fig. 3b). There was also a significant difference between males and females regarding the correlation with age (Fig. 3c). To further explore whether the antibody responses to peptides are gender or age dependent, we performed stratification analysis with age and gender. Our results clearly demonstrated that, on peptide level, we do identify several peptides, i.e., S1-5, S2-59, and S2-82, which are significantly different between males and females (Supplementary Fig. S4i, j). In addition, when we divide the samples into two age groups (< 40 and ≥ 40), significant differences of signals are also observed for several of the peptides, i.e., S1-5, S2-62, S2-82, S2-88, and S2-94 (Supplementary Fig. S4k–l).

We next evaluated whether any peptide is able to differentiate vaccinated volunteers from convalescent and hospitalized patients by comparing signals of the sera collected after the 2nd vaccine dose to those of both patient groups. Indeed, the signals of several peptides were higher in the convalescent group than in the vaccination group, i.e., S1-24 (aa 139–150), S1-113 (aa 673–684), S2-22 (aa 812–823), S2-78 (aa 1148–1159), and S2-94 (aa 1244–1255) (Supplementary Fig. S5a, b). Higher signals for S1-5 (aa 25–36), S2-18 (aa 103–114), S2-23 (aa 818–829), S2-78 (aa 1148–1159), and S2-97 (aa 1262–1273) in the hospitalized patient group than in the vaccination group were also observed (Supplementary Fig. S5c, d). It is notable that both of these comparisons revealed S2-78. As S2-78 has an excellent ability to differentiate COVID-19 patients from non-COVID-19 controls^24^, it may worth further investigation on S2-78 for diagnostic purposes.

### Identification of SARS-CoV-2 proteins and S protein peptides correlating positively with the neutralization activity against the authentic virus

**Fig. 4:**
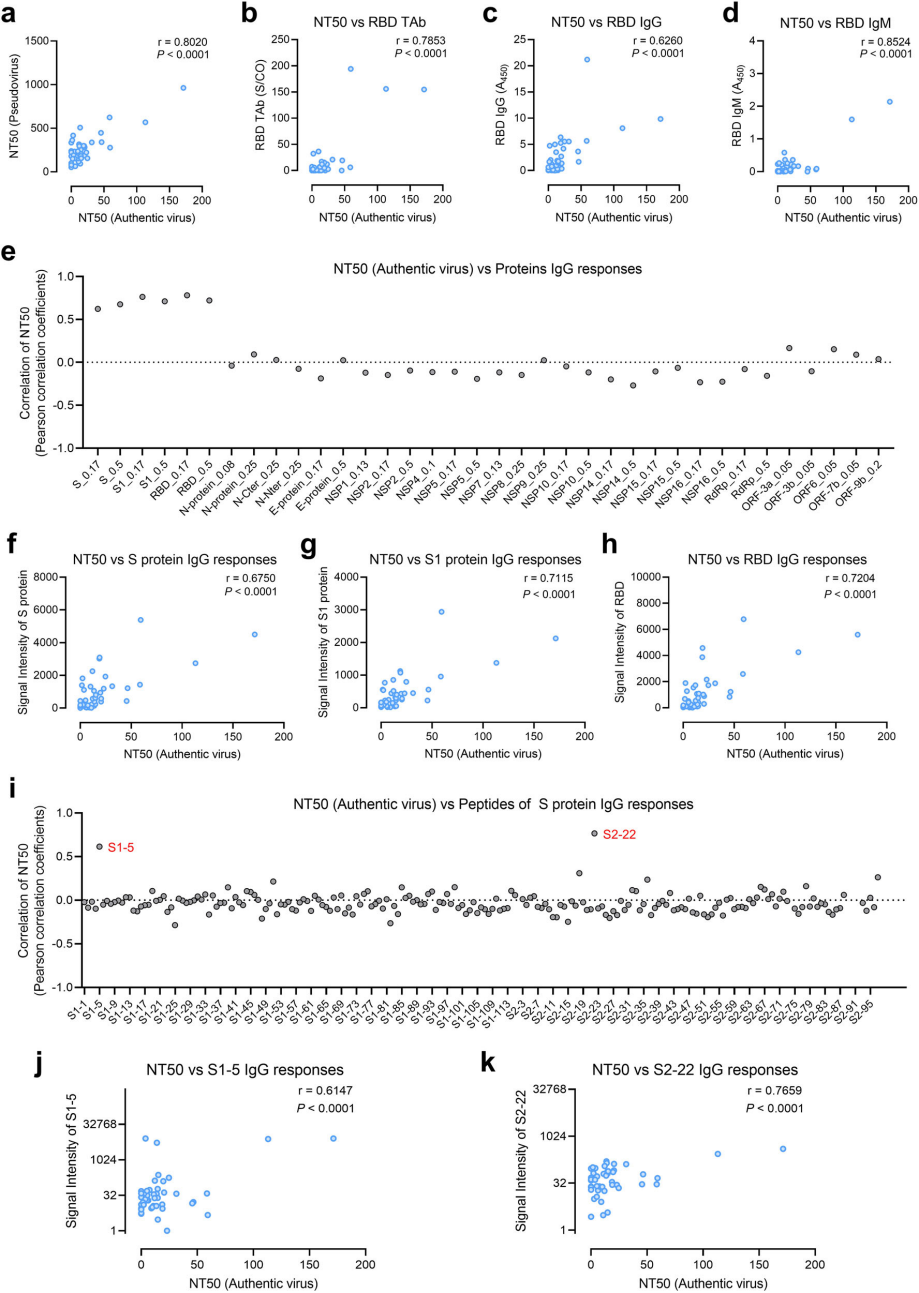
Correlations between neutralization titers (authentic virus) and IgG responses to SARS-CoV-2 proteins or peptides. **a** Correlation of the NT50 (authentic virus) and NT50 (pseudovirus). **b**–**d** Correlations of the NT50 (authentic virus) to ELISA-based assays, i.e., anti-RBD total antibody (TAb, i.e., IgG, IgM, and IgA) (**b**), anti-RBD IgG (**c**), and anti-RBD IgM (**d**). **e** Correlation coefficients for the NT50 (authentic virus) and IgG responses to the SARS-CoV-2 proteins. **f**–**h** Correlation of the NT50 (authentic virus) to IgG responses against the S protein (**f**), S1 subunit (**g**), and RBD (**h**). **i**–**k** Correlation of the NT50 (authentic virus) to IgG responses to all the 197 peptides (**i**), and specifically to S1-5 (**j**), and S2-22 (**k**).

To develop a surrogate biomarker for accurate, easy, and low-cost estimation of the effectiveness of neutralizing antibodies after large-scale vaccination, sera from vaccinated volunteers (Fig. 1b) were subjected to a series of assays, including the neutralization assay of pseudovirus^32^ and enzyme-linked immunosorbent assay (ELISA) measurements of anti-RBD total antibody (TAb, including IgG, IgM, and IgA), anti-RBD IgG, and anti-RBD IgM^33^ (Supplementary Table S1). Neutralization assay with the authentic virus is the gold standard, which was also performed^1^ (Supplementary Table S2). The correlation coefficients between NT50 (authentic virus) and other four assays, i.e., NT50 (pseudovirus), anti-RBD TAb, anti-RBD IgG, and anti-RBD IgM are 0.8020, 0.7853, 0.6260, and 0.8524, respectively. The results of the above assays all correlated well with that of the gold standard (Fig. 4a–d). Our goal was to identify proteins and peptides with the best performance that might serve as surrogate biomarkers. To this end, we performed correlation analyses of neutralization assays of the authentic virus and proteins (Fig. 4e). As expected, good correlations were identified for the S protein (Fig. 4f), S1 (Fig. 4g), and RBD (Fig. 4h), and the correlation coefficients between NT50 (authentic virus) and signal of S protein, S1, and RBD are 0.6750, 0.7115, and 0.7204, respectively. We also performed correlation analyses of the neutralization assays of authentic virus and all S protein peptides (Fig. 4i); interestingly, high correlations were revealed for S1-5 (aa 25–36) (Fig. 4j) and S2-22 (aa 812–823) (Fig. 4k), and the correlation coefficients between NT50 (authentic virus) and signal of S1-5 and S2-22 are 0.6147 and 0.7659, respectively. S1-5 is located at NTD of spike protein and S2-22 is located at the fusion peptide region on S2 subunit of spike protein.

Next, we sought to determine whether it is possible to identify a panel with a higher correlation to the gold standard through the combination of specific proteins and peptides. We started with S, S1, RBD, S1-5, and S2-22 and performed multiple linear regression analysis, revealing panels with correlation values of ~0.9 (Supplementary Fig. S6a), such as S1 + S2-22 (0.0256 × signal intensity of S1 + 0.2403 × signal intensity of S2-22, –7.6518) and RBD + S2-22 (0.0096 × signal intensity of RBD + 0.2406 × signal intensity of S2-22, –7.0404) (Supplementary Fig. S6b).

### Identification of SARS-CoV-2 proteins and S protein peptides that can differentiate vaccinated volunteers from COVID-19 patients

Although the majority of the current immunological assays target either the S or the N protein^34^, inactivated virus vaccines target all the proteins of the virus. Thus, it is necessary to re-evaluate these assays or develop new immunological assays to distinguish vaccinated people from COVID-19 patients.

**Fig. 5:**
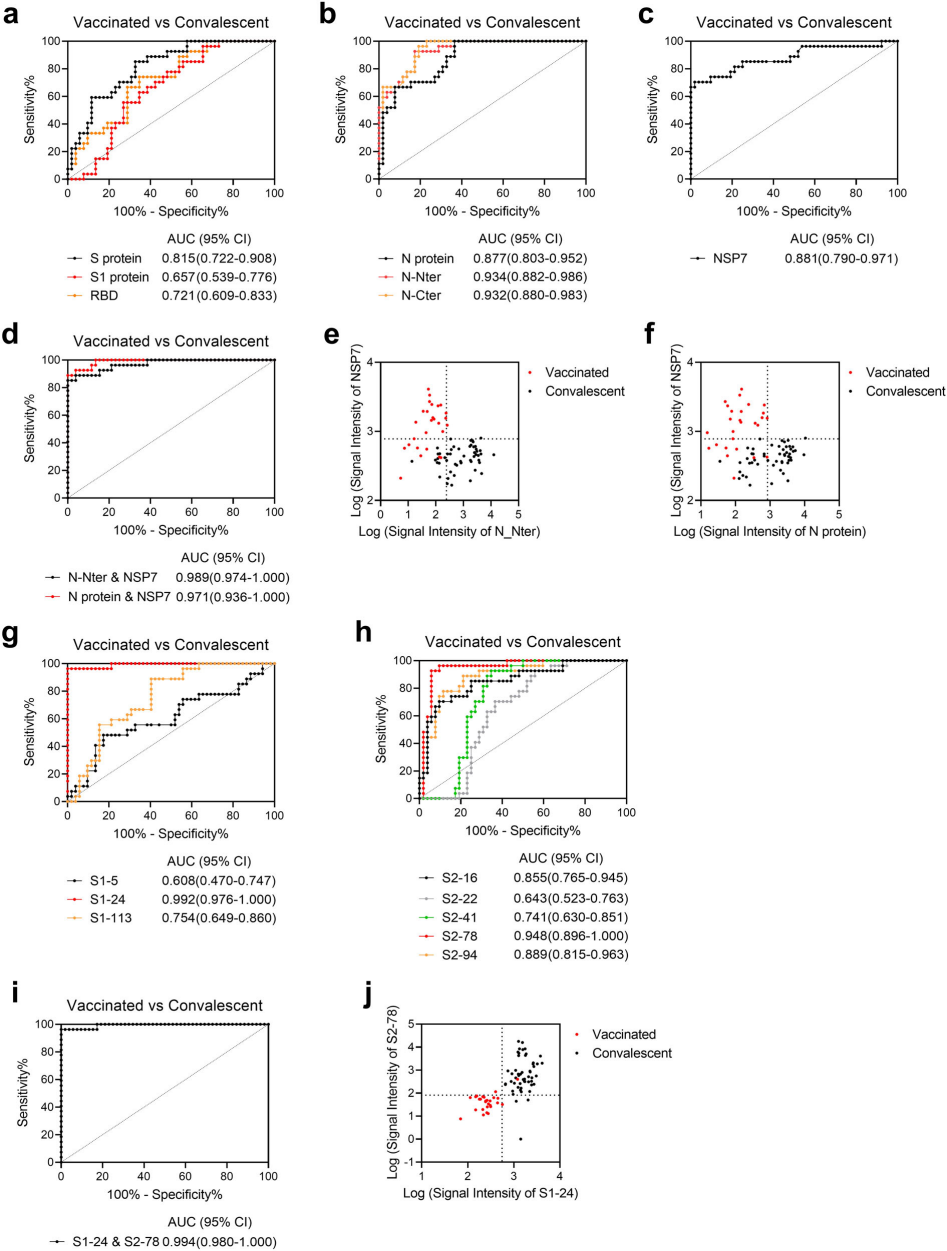
Representative proteins and peptides for differentiating vaccinated volunteers from convalescent patients. **a**–**d** ROC analysis of IgG responses to the S protein (black), S1 subunit (red), and RBD (orange) for comparing vaccinated volunteers and convalescent patients (**a**), the N protein (black), N-Nter (red), and N-Cter (orange) (**b**), NSP7 (**c**), and two combinations, i.e., N-Nter and NSP7 (black) and the N protein and NSP7 (red) (**d**). **e**, **f** Scatter plots of IgG responses of vaccinated volunteers (red dots) and convalescent patients (black dots) for N-Nter vs NSP7 (**e**) and the N protein (**f**) vs NSP7. **g**–**i** ROC analysis of IgG responses to S1-5 (black), S1-24 (red), and S1-113 (orange) for comparing vaccinated volunteers and convalescent patients (**g**), S2-16 (black), S2-22 (gray), S2-41 (green), S2-78 (red), and S2-94 (orange) (**h**), and the combination of S1-24 and S2-78 (**i**). **j** Scatter plots of IgG responses of vaccinated volunteers (red dots) and convalescent patients (black dots) for S1-24 vs S2-78. The gray lines in (**e**), (**f**), and (**j**) indicate cutoff values based on optimal Youden indices of related ROC curves. Unless otherwise stated, sera collected after the 2nd dose were analyzed.

We performed receiver operating characteristic (ROC) analysis to differentiate vaccinated volunteers from convalescent patients. For individual proteins, high area under the curve (AUC) values were obtained for N-Nter (0.934), N-Cter (0.932), NSP7 (0.881), and N (0.877) (Fig. 5a–c). Moreover, the highest AUC value was achieved by combining N-Nter and NSP7 (0.989) (Fig. 5d). In addition, scatter plots showed that the combinations of N-Nter and NSP7, and N and NSP7 were clearly able separate vaccinated volunteers from convalescent patients (Fig. 5e, f). Among the individual peptides, high AUC values were obtained for S1-24 (aa 139–150, 0.992) and S2-78 (aa 463–474, 0.948) in convalescent patients (Fig. 5g, h). The highest AUC value was achieved by combining S1-24 and S2-78 (0.994) (Fig. 5i), and the scatter plots in Fig. 5j illustrate that this combination clearly separates vaccinated volunteers from convalescent patients.

ROC analysis was also performed to differentiate vaccinated volunteers from hospitalized patients. Among the individual proteins, high AUC values were obtained for N-Nter (0.933), N (0.923), and NSP7 (0.819) (Supplementary Fig. S7a, b), and the highest AUC value was achieved by combining N-Nter and N-Cter (0.996) (Supplementary Fig. S7c). Based on scatter plots, the combinations of N-Nter and N-Cter, and N-Nter and NSP7 were clearly able to separate vaccinated volunteers from hospitalized patients (Supplementary Fig. S7d, e). The second-highest AUC value was also found by combining N and N-Cter (0.985) (Supplementary Fig. S7f), and scatter plots showed that the combinations of N and N-Cter, N and NSP7 clearly separated vaccinated volunteers from hospitalized patients (Supplementary Fig. S7g, h). For individual peptides, high AUC values were obtained for S2-78 (aa 1148–1159, 0.930) and S1-105 (aa 625–636, 0.876) (Supplementary Fig. S7i, j), and the highest AUC value was achieved by combining S1-105 and S2-78 (0.930) (Supplementary Fig. S7k). The scatter plots in Supplementary Fig. S7l illustrate that this combination clearly separated vaccinated volunteers from hospitalized patients.

## Discussion

By taking advantage of the SARS-CoV-2 protein microarray and S protein peptide microarray, we in this study generated the first SARS-CoV-2-specific global antibody response profile for vaccination with an inactivated virus vaccine. We detected obvious differences by comparing this profile to those of convalescent and hospitalized patients. Moreover, several proteins and peptides that have the potential to predict the effectiveness of vaccination and to differentiate vaccinated individuals from convalescent and hospitalized patients were identified.

At the protein level, the overall profile of IgG responses after the 2nd vaccine dose was similar to that observed in convalescent patients. Even at 28 days after the 1st dose, the IgG signals of S, S1, RBD, N, N-Nter, and N-Cter in volunteers were significantly lower than those in the convalescent group. Because neutralizing antibodies primarily target the S protein, especially the RBD^27,28^, and SARS-CoV-2-specific T cell responses induced by the inactivated virus vaccine are weak^16^, the significantly enhanced IgG responses after the second vaccine dose may serve as evidence of the necessity of the two-dose vaccination strategy for the inactivated virus vaccine. It is worth noting that even after the 2nd dose, the signals of S, S1, and RBD in the vaccination group were lower than those in the convalescent group. This indicates a weaker IgG response elicited by the inactivated virus vaccine than that of real infection, which is consistent with the results for CoronaVac^29^. However, as the IgG response is an overall reflection of antibody level and affinity, the factor(s) that cause the IgG response differences between vaccination and infection need to be further explored. In addition, the signal of N, N-Nter, and N-Cter were also significantly lower for the vaccinated people than those in the convalescent group. The signal of N-Cter is higher than N-Nter, it maybe because that the N-Cter is immunodominant. Indeed, we have mapped the epitopes on N protein by using a high-throughput epitope mapping technology (AbMap), and the region spans from aa 363 to 416, and is highly immunodominant and belongs the C-terminal of N protein^35^.

Nevertheless, the significantly higher NSP7 signal in the vaccinated group than in the other two groups was unexpected. More interestingly, IgG antibody levels of NSP7, NSP8, and NSP9 were consistent among all four time points after vaccination. NSP7 may form a complex with NSP8, and RdRp participates in viral replication by acting as a primase^36^. Hence, NSP7/NSP8/RdRp elicits profound IgG responses shortly after the 1st vaccine dose. One plausible explanation is that pre-existing memory B cells secrete antibodies that specifically recognize epitopes of NSP7/NSP8/RdRp. Indeed, most of the healthy controls examined exhibited positive, though weak, signals for NSP7 but not for the majority of the other SARS-CoV-2 proteins on the microarray (Fig. 1c). This hypothesis and the functional roles of NSP7/NSP8/RdRp-specific antibodies warrant further investigation.

The overall profile of IgG responses was similar to that of hospitalized patients at all four time points, except for the S and N proteins. IgG signals for the S and N proteins were still very weak at 28 days after the 1st vaccine dose, though significantly high signals were observed for patient sera collected at Time 2. This difference may be explained by the distinct natures of the S and N proteins of inactivated and live viruses.

Although S, S1, and RBD IgG signals in the vaccination group were slightly higher in females than in males, the difference was not significant, which was similar to the trend observed for convalescent patients^22,31^. These results indicate that the effectiveness of inactivated virus vaccination is similar between males and females. Interestingly, no difference between males and females was also reported in a study on the inactivated virus vaccine CoronaVac^29^. For both males and females, the IgG responses to S, S1, and RBD correlated negatively with age. It is known that older individuals usually have lower immunity, and a plausible explanation for the negative correlation is that levels of IgG responses to S, S1, and RBD to some extent reflect immunity. Interestingly, according to our previous study^22^ and others^28,31^, IgG responses correlate positively with age in COVID-19 patients. This inconsistency may be explained by differences in immune responses to the inactivated virus and the live virus.

At the peptide level, overall IgG signals in the vaccination group were also significantly lower than those in both the convalescent and hospitalized groups. This further confirmed that the IgG responses elicited by the inactivated virus vaccine were weaker than those elicited by the real infection. Interestingly, the pattern of IgG responses to the inactivated virus vaccine also differed significantly from those of convalescents and hospitalized patients^23,25^. This may be due to differences in the presentation and duration of the S protein between the inactivated and live viruses. It is worth noting that S1-61 gives high signal in healthy volunteers. S1-61 is located at RBD region, and the sequence is CVADYSVLYNSA (aa 361–372). The high signals of S1-61 on healthy volunteers may be due to the cross-reaction caused by another and more generic infection, which is highly prevalent. The underlying mechanism warrants further investigation.

None of the current SARS-CoV-2 vaccines is 100% effective or strong enough to provide protection immediately after vaccination. For example, the effectiveness of the inactivated virus vaccine BBIBP-CorV is ~78.1%^20^. The question here is who have been protected by the vaccine and who have not. Theoretically, this might be addressed by measuring the neutralization activities of sera collected from vaccinated individuals using the authentic virus. However, this is practically impossible due to the limited availability of biosafety facilities. Other approaches include pseudovirus neutralization assays^37,38^ and assays of anti-RBD TAb, anti-RBD IgG, and anti-RBD IgM^29,33^. In this study, all four assays showed good correlation with the authentic virus assay, and anti-RBD TAb test could be an effective way to evaluate the efficacy of inactivated vaccine. The limitations of these assays include requirements of sophisticated experimental operations and the preparation of active proteins.

In this study, we identified two peptides with high correlations to the authentic virus assay, i.e., S1-5 and S2-22. When combining these two peptides, especially S2-22, with S, S1, or RBD, high correlation values of ~0.9 were obtained. In comparison to the protein-based assays, peptide-based assays have the following advantages: low cost of synthesis, high purity, and high stability at various temperatures. These two peptides might be applied independently or in combination with proteins to develop surrogate assays for accurate, easy, and low-cost estimation of the effectiveness of inactivated virus vaccination. One ideal assay is the lateral strip assay^39,40^ accompanied by a portable device, enabling self-administration of the test at home, similar to the blood glucose test^41^.

Regarding COVID-19 diagnostics, immunological assays are complementary to nucleic acid tests (NATs), whereas immunological assays are the only practical choice for other applications, e.g., assessing prevalence. As inactivated viruses retain intact viral particles with the full-length S and N proteins, the current S and N protein-based immunological assays may not be suitable after large-scale vaccination. To address this challenge, we found that at the protein level, N protein is still capable of differentiating vaccinated people from both convalescent and hospitalized patients; its derivative N-Nter showed the best performance. This indicates that the approved N protein-based immunological assay may still be applicable after large-scale vaccination, but a new cutoff needs to be carefully set. The most interesting finding is that NSP7 demonstrated good differentiation performance. At the peptide level, peptide S2-78 performed the best at differentiating vaccinated individuals from both convalescent and hospitalized patients. Interestingly, S2-78 also performs well in differentiating patients from non-COVID-19 controls^24^. After careful assay development and further validation with a large sample cohort, the N protein, NSP7, and S2-78 may be applied independently or in combination for effective immunological assays during vaccination campaigns and in the coming post-vaccination era.

It should be noted that only one inactivated virus vaccine (BBIBP-CorV) was included in this study. Nonetheless, the basic manufacturing protocols for different inactivated vaccines, e.g., CoronaVac^29^ and the inactivated COVID-19 vaccine from the Wuhan Institute of Biological Products^18^, are similar. Thus, we anticipate that the findings of this study will also be applicable to other inactivated vaccines after slight adjustments.

Taken together, the results provide a comprehensive antibody profile for the inactivated virus vaccine BBIBP-CorV, which may facilitate an in-depth understanding of the humoral immunity of the vaccine at the systemic level. With extensive validation on a large cohort of samples, we believe that potential surrogate biomarker panels can be applied for assessing the effectiveness of vaccination and differentiating vaccinated individuals from patients.

## Materials and methods

### Samples

Informed consent was obtained from all vaccinated volunteers enrolled in studies at the Beijing BGI Clinical Laboratories. In addition, the study conformed to the principles of the Declaration of Helsinki. The IRB of BGI-Shenzhen approved the microarray-based serum analysis and related downstream analyses of samples collected by the aforementioned institution under ethical clearance No. BGI-IRB 20158. The interval between the two doses was 28 days, and all sera were stored at −80 °C until use.

The Institutional Ethics Review Committee of Foshan Fourth Hospital in Foshan, China, approved this study, and written informed consent was obtained from each patient. COVID-19 patients were hospitalized and received treatment at Foshan Fourth Hospital from January 25, 2020 to February 27, 2020; the patients were hospitalized for variable amounts of time. Serum from each patient was collected on the day of hospital discharge, when the standard criteria were met according to the Diagnosis and Treatment Protocol for Novel Coronavirus Pneumonia (Trial Version 5) released by the National Health Commission & State Administration of Traditional Chinese Medicine. All sera were stored at −80 °C until use.

### Serological tests

Total antibody (TAb), IgG, and IgM levels against the RBD were detected by using chemiluminescent immunoassay (CLIA) and ELISA kits produced by Beijing Wantai Biological Pharmacy Enterprise Co., Ltd., and the experiments were performed according to the manufacturer’s instructions. In brief, the TAb response to the RBD was detected by double-antigen sandwich CLIA using the RBD and HRP-conjugated RBD. IgG responses to the RBD were detected by indirect ELISA, and IgM responses to the RBD were detected by the IgM μ-chain capture method. IgG samples with an A_450_ to cutoff ratio higher than 10 were further gradient diluted (1:15, 1:45, 1:135) and tested again, and the titer was calculated by multiplying A_450_ by the maximum dilution factor.

### Microarray-based serum analysis

Microarray analysis was conducted as described previously^22^. Briefly, arrays were blocked with 3% BSA-PBS buffer for 3 h, and a 14-chamber rubber gasket was mounted onto each slide. Then, 200 μL of serum diluted 1:200 with 1% BSA-PBST (0.1% Tween-20) was added to the subarray and incubated at room temperature for 2 h. The arrays were washed 3 times with PBST and incubated with Cy3-conjugated goat anti-human IgG and Alexa Fluor 647-conjugated donkey anti-human IgM at room temperature for 1 h. The arrays were washed 3 times with PBST, dried by centrifugation, and scanned using a LuxScan 10K-A instrument (CapitalBio Corporation, Beijing, China) with 100% laser power and PMT 450. The signal intensity was extracted by GenePix Pro 6.0 software (Molecular Devices, CA, USA).

### Neutralization assay of pseudovirus

A pseudovirus incorporating the spike protein was constructed, and a neutralization assay was conducted as described previously^42^. In brief, 100 μL serial dilutions of sera from volunteers were mixed with 50 μL of pseudovirus (1300 TCID_50_/mL) in plates and incubated at 37 °C for 1 h; 2 × 10^4^/100 μL Huh-7 cells were then added to the plates and incubated at 37 °C and 5% CO_2_ for 24 h. Chemiluminescence detection was performed, and the Reed-Muench method was used to calculate the NT50.

### Neutralization assay of the authentic virus

A plaque reduction neutralization test (PRNT) was performed to detect the NT50 of volunteer serum samples. Vero-E6 cells were grown at 37 °C and 5% CO_2_ for 24 h, and 300 μL serial dilutions of sera from volunteers were mixed with an equal volume of authentic SARS-CoV-2 virus (300 PFU/mL) and incubated at 37 °C for 1 h. Next, 500 μL serum–virus mixture was added to Vero-E6 cells. After incubation at 37 °C for 1 h, the culture medium of the serum–virus mixture was replaced with 2.5% FBS-DMEM containing 0.8% carboxymethylcellulose, and the mixture was further incubated at 37 °C and 5% CO_2_ for 4 days. The cells were then fixed with 8% paraformaldehyde and stained with 0.5% crystal violet. Plaques were counted, the inhibition rate was calculated, and the NT50 was determined by normalized response logistic regression analysis using GraphPad Prism 9.0.

### Quantification and statistical analysis

For the microarray, signal intensity was defined as the median of the foreground subtracted by the median of the background, and the signal intensities of triplicate spots of each peptide protein were averaged. GraphPad Prism 9.0 was used for the plotting and logistic regression, Student’s *t*-test, two-way analysis of variance (ANOVA), Pearson correlation, and ROC curve statistical analyses. Statistical Product and Service Solutions (SPSS) software was applied for multiple linear regression.

## Supplementary information

Supplementary information.

## Data Availability

The microarray data generated during this study were deposited at Protein Microarray Database (http://www.proteinmicroarray.cn) under accession number PMDE250.
