# Supplementary material for: Systematic profiling of SARS-CoV-2-specific IgG responses elicited by an inactivated virus vaccine identifies peptides and proteins for predicting vaccination efficacy
Source: Cell Discov. 2021 Aug 17;7:67. doi: 10.1038/s41421-021-00309-7 (PMC8367966; doi:10.1038/s41421-021-00309-7)
Supplement: Supplementary file 1 — Supplementary information. [file 41421_2021_309_MOESM1_ESM.pdf]

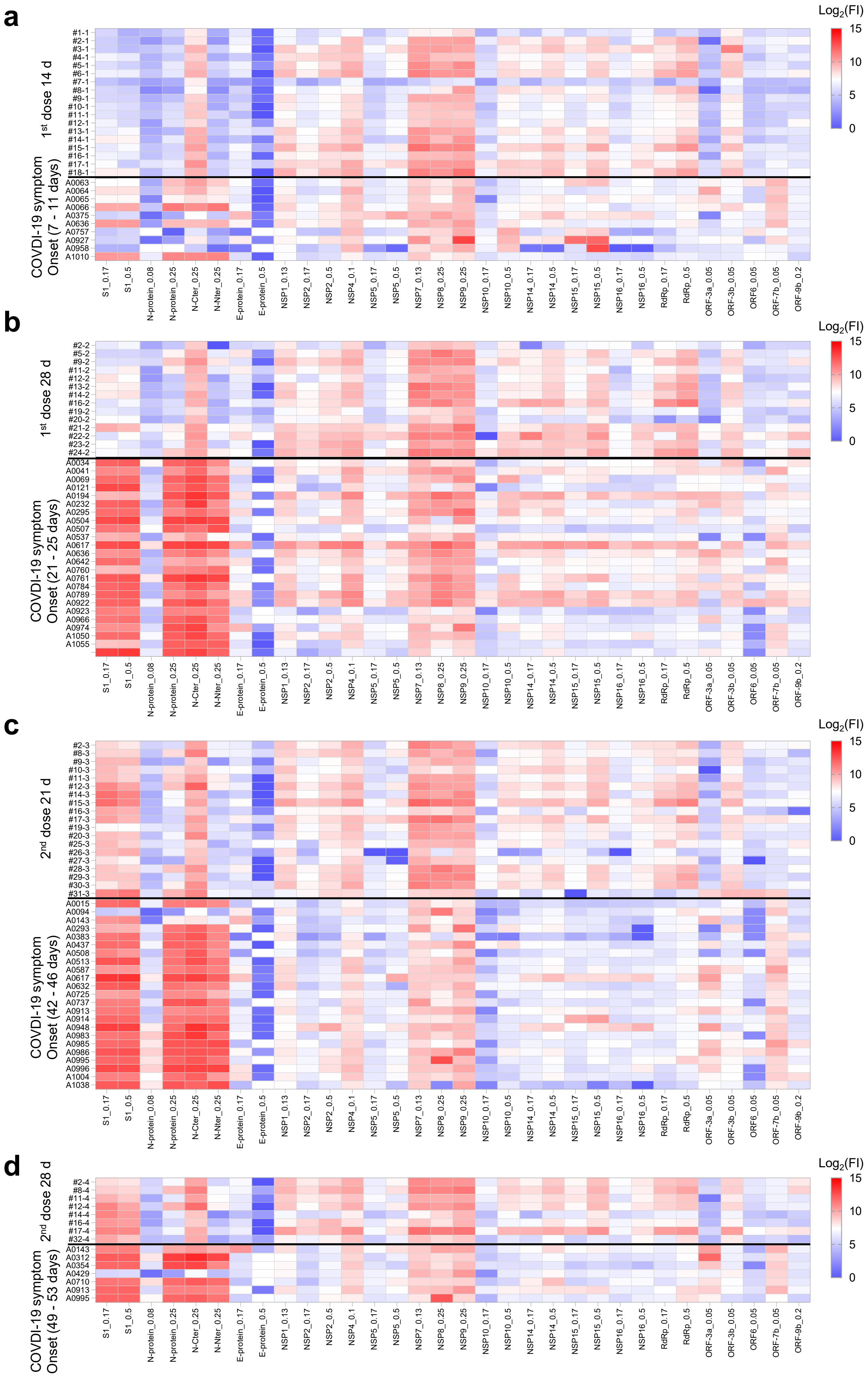
a
1st dose 14 d
COVDI-19 symptom Onset (7 - 11 days)
#1-1
#2-1
#3-1
#4-1
#5-1
#6-1
#7-1
#8-1
#9-1
#10-1
#11-1
#12-1
#13-1
#14-1
#15-1
#16-1
#17-1
#18-1
A0063
A0064
A0065
A0066
A0375
A0636
A0757
A0927
A0958
A1010
S1_0.17
S1_0.5
N-protein_0.08
N-protein_0.25
N-Cter_0.25
N-Nter_0.25
E-protein_0.17
E-protein_0.5
NSP1_0.13
NSP2_0.17
NSP2_0.5
NSP4_0.1
NSP5_0.17
NSP5_0.5
NSP7_0.13
NSP8_0.25
NSP9_0.25
NSP10_0.17
NSP10_0.5
NSP14_0.17
NSP14_0.5
NSP15_0.17
NSP15_0.5
NSP16_0.17
NSP16_0.5
RdRp_0.17
RdRp_0.5
ORF-3a_0.05
ORF-3b_0.05
ORF6_0.05
ORF-7b_0.05
ORF-9b_0.2
Log_2(FI)
15
10
5
0
b
1st dose 28 d
COVDI-19 symptom Onset (21 - 25 days)
#2-2
#5-2
#9-2
#11-2
#12-2
#13-2
#14-2
#16-2
#19-2
#20-2
#21-2
#22-2
#23-2
#24-2
A0034
A0041
A0069
A0121
A0194
A0232
A0295
A0504
A0507
A0537
A0617
A0636
A0642
A0760
A0761
A0784
A0789
A0922
A0923
A0966
A0974
A1050
A1055
S1_0.17
S1_0.5
N-protein_0.08
N-protein_0.25
N-Cter_0.25
N-Nter_0.25
E-protein_0.17
E-protein_0.5
NSP1_0.13
NSP2_0.17
NSP2_0.5
NSP4_0.1
NSP5_0.17
NSP5_0.5
NSP7_0.13
NSP8_0.25
NSP9_0.25
NSP10_0.17
NSP10_0.5
NSP14_0.17
NSP14_0.5
NSP15_0.17
NSP15_0.5
NSP16_0.17
NSP16_0.5
RdRp_0.17
RdRp_0.5
ORF-3a_0.05
ORF-3b_0.05
ORF6_0.05
ORF-7b_0.05
ORF-9b_0.2
Log_2(FI)
15
10
5
0
c
2nd dose 21 d
COVDI-19 symptom Onset (42 - 46 days)
#2-3
#8-3
#9-3
#10-3
#11-3
#12-3
#14-3
#15-3
#16-3
#17-3
#19-3
#20-3
#25-3
#26-3
#27-3
#28-3
#29-3
#30-3
#31-3
A0015
A0094
A0143
A0293
A0383
A0437
A0508
A0513
A0587
A0617
A0632
A0725
A0737
A0913
A0914
A0948
A0983
A0985
A0986
A0995
A0996
A1004
A1038
S1_0.17
S1_0.5
N-protein_0.08
N-protein_0.25
N-Cter_0.25
N-Nter_0.25
E-protein_0.17
E-protein_0.5
NSP1_0.13
NSP2_0.17
NSP2_0.5
NSP4_0.1
NSP5_0.17
NSP5_0.5
NSP7_0.13
NSP8_0.25
NSP9_0.25
NSP10_0.17
NSP10_0.5
NSP14_0.17
NSP14_0.5
NSP15_0.17
NSP15_0.5
NSP16_0.17
NSP16_0.5
RdRp_0.17
RdRp_0.5
ORF-3a_0.05
ORF-3b_0.05
ORF6_0.05
ORF-7b_0.05
ORF-9b_0.2
Log_2(FI)
15
10
5
0
d
2nd dose 28 d
COVDI-19 symptom Onset (49 - 53 days)
#2-4
#8-4
#11-4
#12-4
#14-4
#16-4
#17-4
#32-4
A0143
A0312
A0354
A0429
A0710
A0913
A0995
S1_0.17
S1_0.5
N-protein_0.08
N-protein_0.25
N-Cter_0.25
N-Nter_0.25
E-protein_0.17
E-protein_0.5
NSP1_0.13
NSP2_0.17
NSP2_0.5
NSP4_0.1
NSP5_0.17
NSP5_0.5
NSP7_0.13
NSP8_0.25
NSP9_0.25
NSP10_0.17
NSP10_0.5
NSP14_0.17
NSP14_0.5
NSP15_0.17
NSP15_0.5
NSP16_0.17
NSP16_0.5
RdRp_0.17
RdRp_0.5
ORF-3a_0.05
ORF-3b_0.05
ORF6_0.05
ORF-7b_0.05
ORF-9b_0.2
Log_2(FI)
15
10
5
0

Supplementary Figure S1. Comparison of SARS-CoV-2-specific IgG responses between vaccinated volunteers and hospitalized patients.

a-d IgG responses of vaccinated volunteers and hospitalized patients against the SARS-CoV-2 proteins. Sera analyzed in each panel were collected at different time points, i.e., 14 days after the 1st vaccine dose and 7-11 days after COVID-19 symptom onset (**a**), 28 days after the 1st vaccine dose and 21-25 days after COVID-19 symptom onset (**b**), 14 days after the 2nd vaccine dose and 42-46 days after COVID-19 symptom onset (**c**) and 28 days after the 2nd vaccine dose and 49-53 days after COVID-19 symptom onset (**d**), for the vaccinated volunteers and hospitalized patients, respectively.

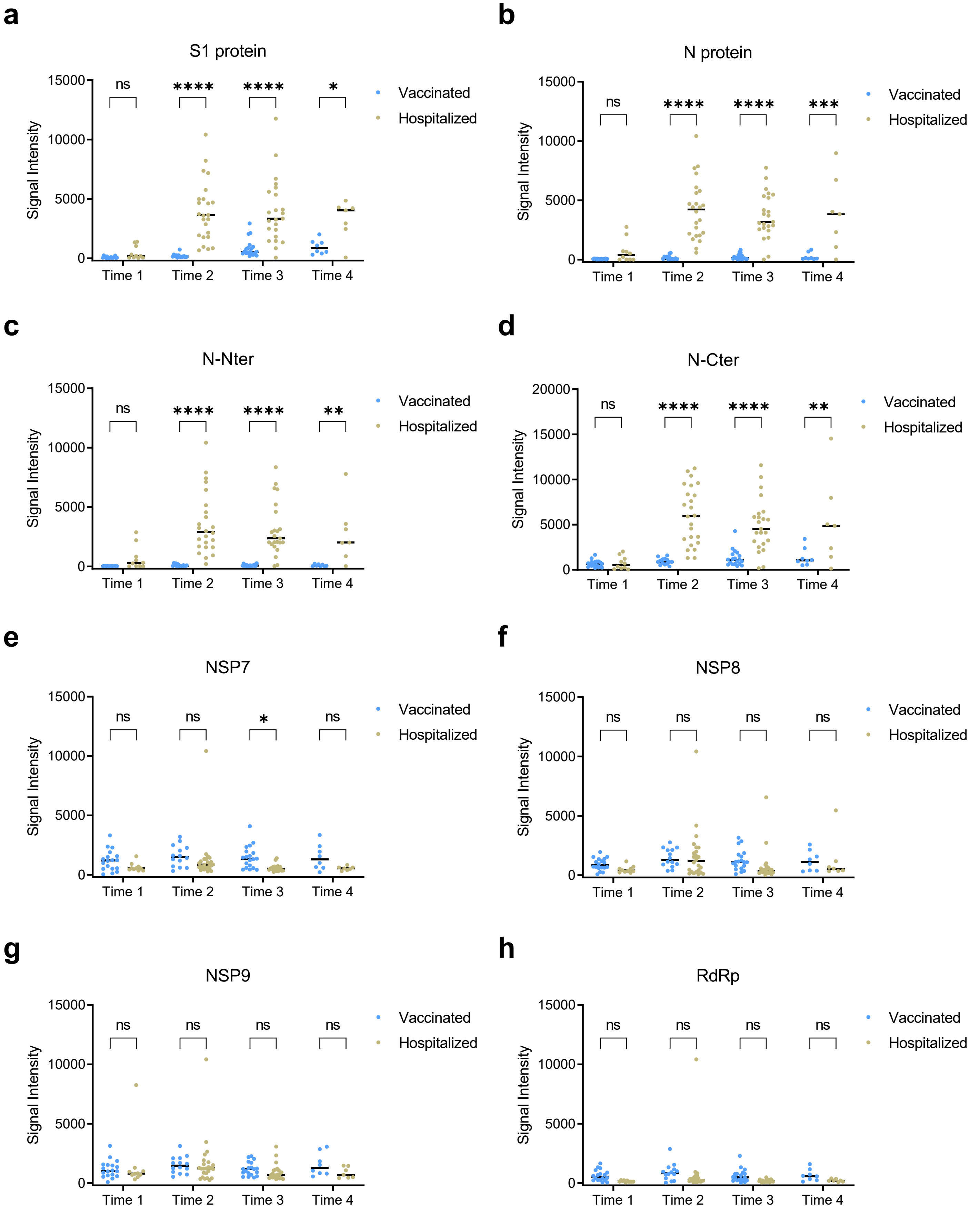

Supplementary Figure S2. Comparison of SARS-CoV-2-specific IgG responses to representative proteins between vaccinated volunteers and hospitalized patients.
a-h IgG responses to S1 (**a**), the N protein (**b**), N-Nter (**c**), N-Cter (**d**), NSP7 (**e**), NSP8 (**f**), NSP9 (**g**) and RdRp (**h**). *P* values were calculated by the Student's *t*-test, *P* value: * < 0.05, ** < 0.01, *** < 0.005, **** < 0.001, ns represents no significant difference. Sera collected at comparable time points from both the immunized volunteers and hospitalized patients were compared.

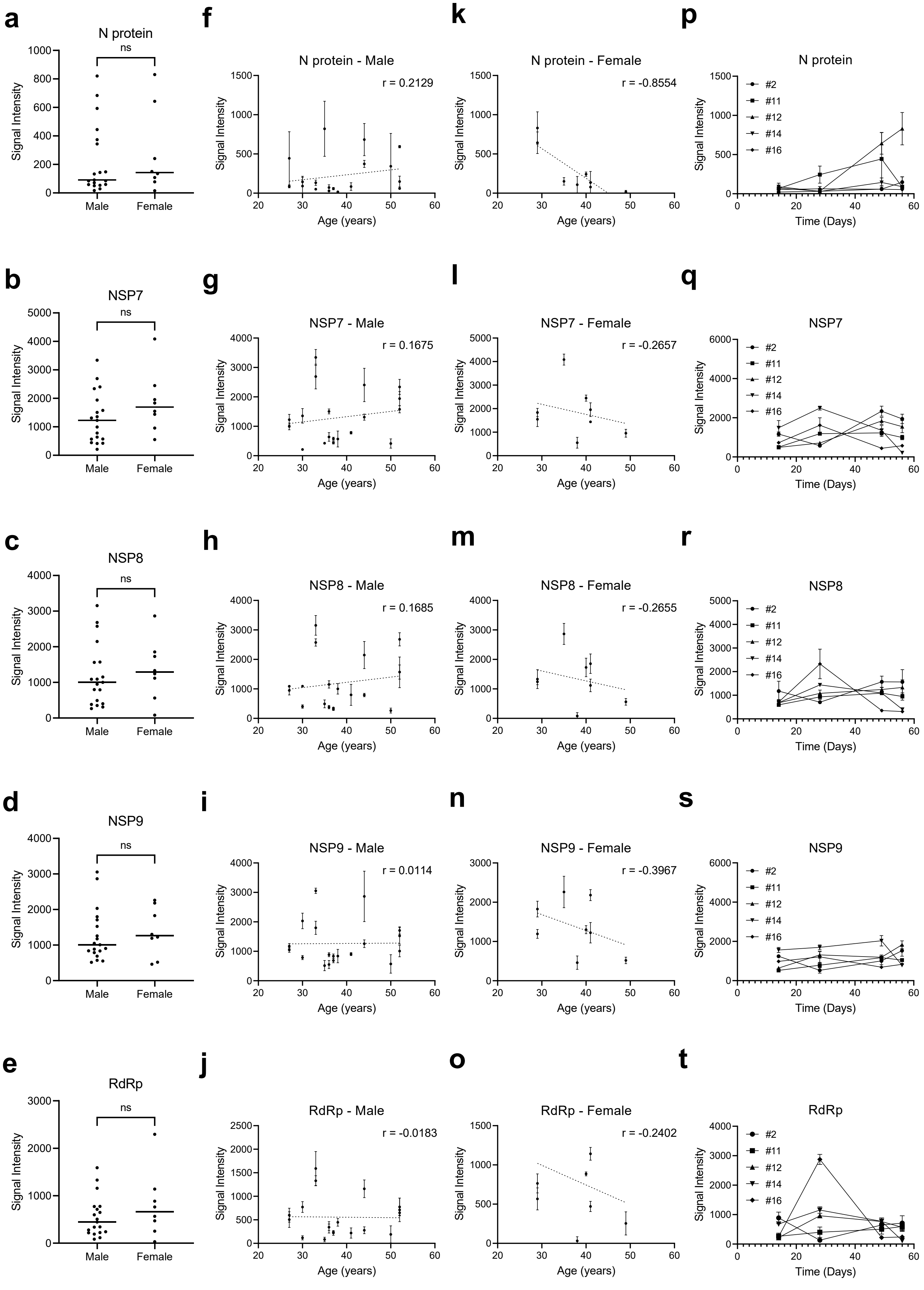

a
N protein
ns
Signal Intensity
Male
Female
f
N protein - Male
r = 0.2129
Age (years)
k
N protein - Female
r = -0.8554
p
N protein
#2
#11
#12
#14
#16
Time (Days)
b
NSP7
g
NSP7 - Male
r = 0.1675
l
NSP7 - Female
r = -0.2657
q
NSP7
c
NSP8
h
NSP8 - Male
r = 0.1685
m
NSP8 - Female
r = -0.2655
r
NSP8
d
NSP9
i
NSP9 - Male
r = 0.0114
n
NSP9 - Female
r = -0.3967
s
NSP9
e
RdRp
j
RdRp - Male
r = -0.0183
o
RdRp - Female
r = -0.2402
t
RdRp

Supplementary Figure S3. Correlation of protein (proteins other than S, S1 and RBD)-specific IgG responses to age or gender.

a-e IgG responses to the N protein (**a**), NSP7 (**b**), NSP8 (**c**), NSP9 (**d**) and RdRp (**e**) in males vs females. *P* values were calculated by the Student's *t*-test. **f-j** Correlations of IgG responses to age (male). IgG responses to the N protein (**f**), NSP7 (**g**), NSP8 (**h**), NSP9 (**i**), and RdRp (**j**). **k-o** Correlations of IgG responses to age (female). IgG responses to the N protein (**k**), NSP7 (**l**), NSP8 (**m**), NSP9 (**n**), and RdRp (**o**). **p-t** Longitudinal IgG responses to the N protein (**p**), NSP7 (**q**), NSP8 (**r**), NSP9 (**s**) and RdRp (**t**). *P* values are presented as the mean ± SEM. Unless otherwise stated, sera collected after the 2nd vaccine dose were analyzed.

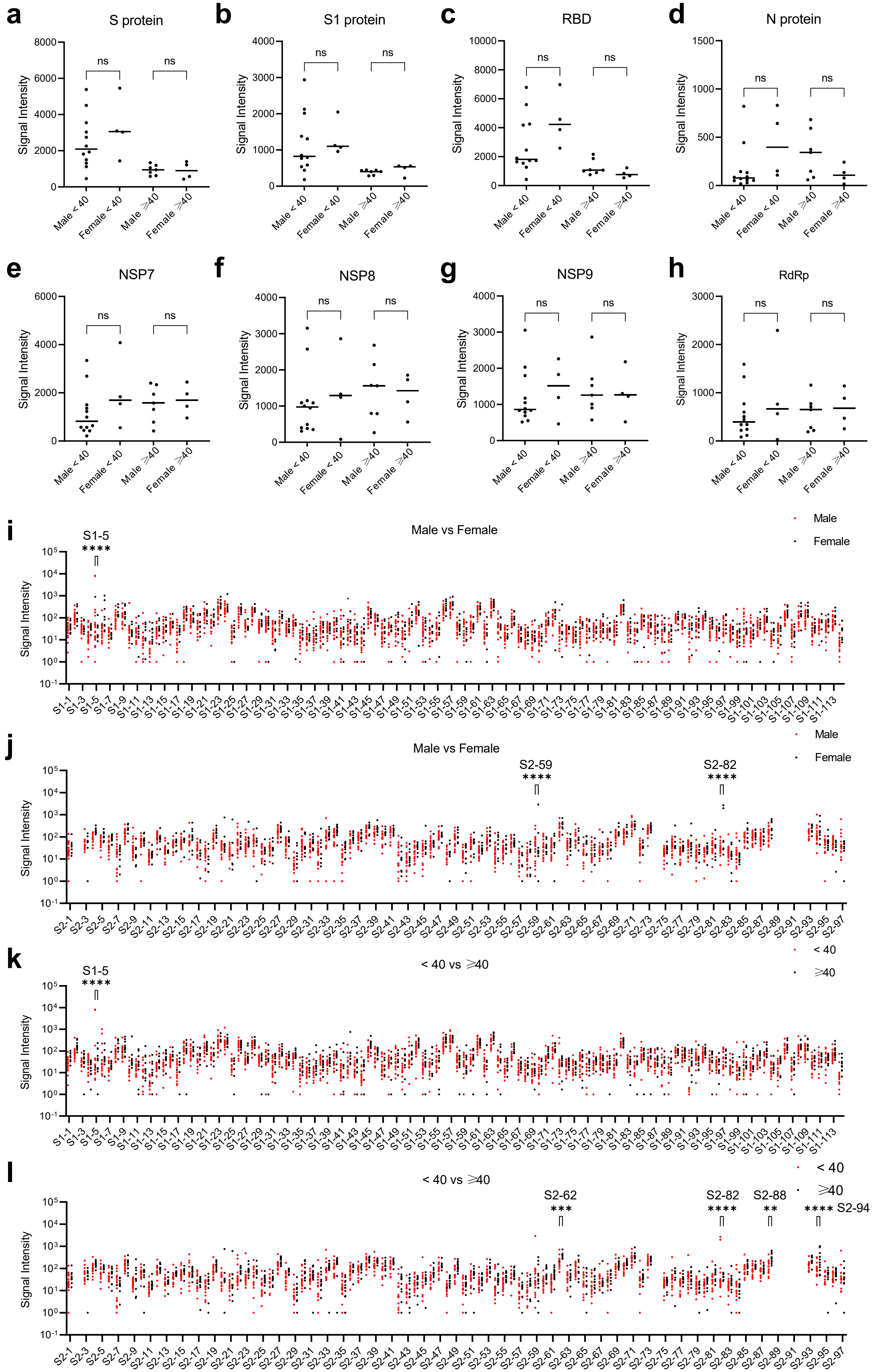
a
S protein
Signal Intensity
ns
ns
Male < 40
Female < 40
Male ⩾40
Female ⩾40
b
S1 protein
c
RBD
d
N protein
e
NSP7
f
NSP8
g
NSP9
h
RdRp
i
Male vs Female
S1-5
****
Male
Female
S1-1 S1-3 S1-5 S1-7 S1-9 S1-11 S1-13 S1-15 S1-17 S1-19 S1-21 S1-23 S1-25 S1-27 S1-29 S1-31 S1-33 S1-35 S1-37 S1-39 S1-41 S1-43 S1-45 S1-47 S1-49 S1-51 S1-53 S1-55 S1-57 S1-59 S1-61 S1-63 S1-65 S1-67 S1-69 S1-71 S1-73 S1-75 S1-77 S1-79 S1-81 S1-83 S1-85 S1-87 S1-89 S1-91 S1-93 S1-95 S1-97 S1-99 S1-101 S1-103 S1-105 S1-107 S1-109 S1-111 S1-113
j
Male vs Female
S2-59
****
S2-82
****
S2-1 S2-3 S2-5 S2-7 S2-9 S2-11 S2-13 S2-15 S2-17 S2-19 S2-21 S2-23 S2-25 S2-27 S2-29 S2-31 S2-33 S2-35 S2-37 S2-39 S2-41 S2-43 S2-45 S2-47 S2-49 S2-51 S2-53 S2-55 S2-57 S2-59 S2-61 S2-63 S2-65 S2-67 S2-69 S2-71 S2-73 S2-75 S2-77 S2-79 S2-81 S2-83 S2-85 S2-87 S2-89 S2-91 S2-93 S2-95 S2-97
k
< 40 vs ⩾40
S1-5
****
< 40
⩾40
l
< 40 vs ⩾40
S2-62
***
S2-82
****
S2-88
**
**** S2-94

Supplementary Figure S4. Comparison of peptides-specific IgG responses between different age or gender.

a-h IgG responses to the S protein (**a**), S1 subunit (**b**), RBD (**c**), N protein (**d**), NSP7 (**e**), NSP8 (**f**), NSP9 (**g**) and RdRp (**h**) of males vs females in two groups (<40 and >=40). The *p*-value were calculated by the Student's *t*-test. **i-j** Comparison of peptides-specific IgG responses between male and female. Peptides of S1 (**i**) and S2 (**j**). **k-l** Comparison of peptides-specific IgG responses between <40 and >=40 groups. Peptides of S1 (**k**) and S2 (**l**). The *P* values were calculated by two-way ANOVA, *P* value: * < 0.05, ** < 0.01, *** < 0.005, and **** < 0.001.

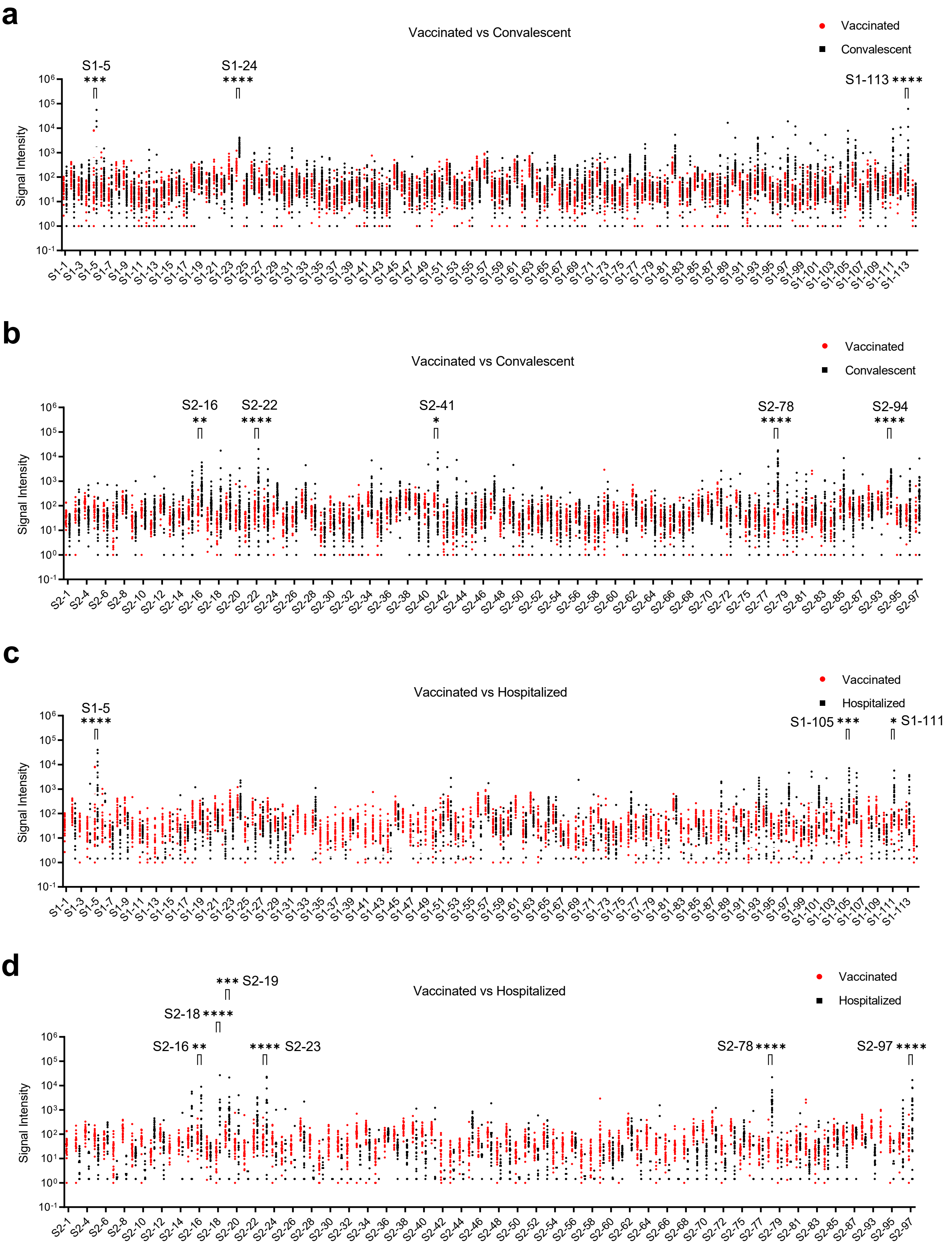

Supplementary Figure S5. Comparison of peptides-specific IgG responses between vaccinated volunteers and convalescent or hospitalized patients.

a-b Comparison of peptides-specific IgG responses between vaccinated volunteers and convalescent patients. Peptides of S1 (**a**) and S2 (**b**). **c-d** Comparison of peptides-specific IgG responses between vaccinated volunteers and hospitalized patients. Peptides of S1 (**c**) and S2 (**d**). *P* values were calculated by two-way ANOVA, *P* value: * < 0.05, ** < 0.01, *** < 0.005, and **** < 0.001.

a

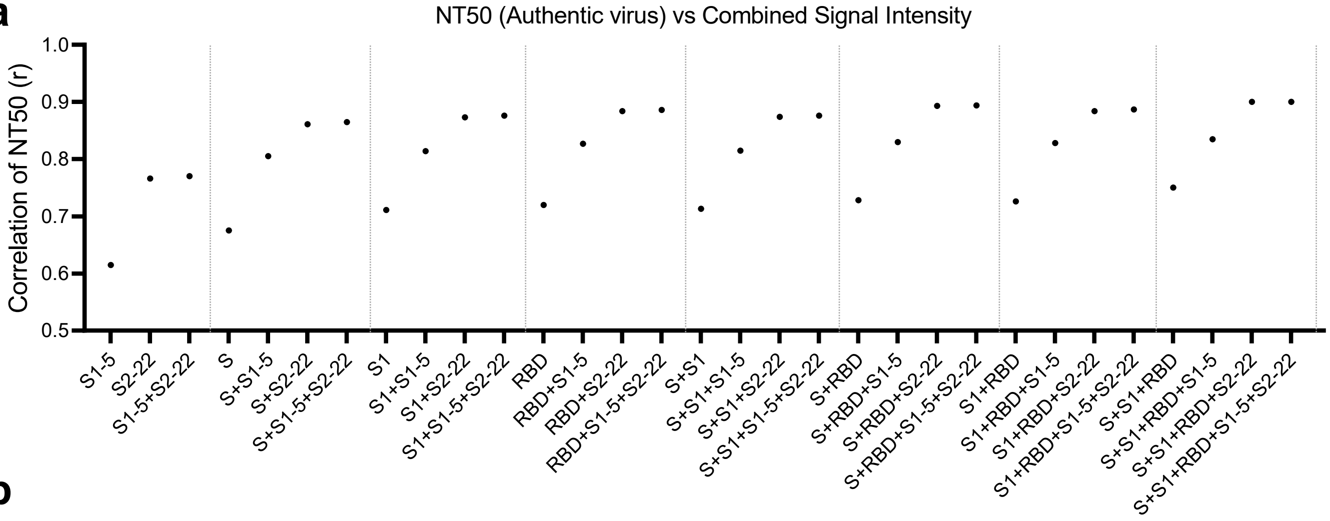

b

Panel	Correlation (r)	Coefficient (S)	Coefficient (S1)	Coefficient (RBD)	Coefficient (S1-5)	Coefficient (S2-22)	Constant
S1-5	0.615	-	-	-	0.0089	-	13.4464
S2-22	0.766	-	-	-	-	0.3271	-1.6493
S1-5+S2-22	0.770	-	-	-	0.0016	0.2923	-0.4977
S	0.675	0.0176	-	-	-	-	2.4911
S+S1-5	0.805	0.0142	-	-	0.0066	-	1.2601
S+S2-22	0.861	-	0.0113	-	-	0.2506	-7.6276
S+S1-5+S2-22	0.865	0.0113	-	-	0.0018	0.2106	-6.3495
S1	0.711	0.0391	-	-	-	-	1.8516
S1+S1-5	0.814	-	0.0312	-	0.0061	-	1.2089
S1+S2-22	0.873	-	0.0256	-	-	0.2403	-7.6518
S1+S1-5+S2-22	0.876	-	0.0255	-	0.0013	0.2123	-6.6799
RBD	0.720	-	-	0.0142	-	-	3.2749
RBD+S1-5	0.827	-	-	0.0115	0.0062	-	2.0733
RBD+S2-22	0.884	-	-	0.0096	-	0.2406	-7.0404
RBD+S1-5+S2-22	0.886	-	-	0.0096	0.0014	0.2099	-5.9997
S+S1	0.713	-0.0045	0.0482	-	-	-	2.0700
S+S1+S1-5	0.815	0.0019	0.0273	-	0.0062	-	1.1112
S+S1+S2-22	0.874	-0.0033	0.0323	-	-	0.2400	-7.4782
S+S1+S1-5+S2-22	0.876	-0.0021	0.0299	-	0.0012	0.2140	-6.6343
S+RBD	0.728	-0.0112	-	0.0224	-	-	4.7070
S+RBD+S1-5	0.830	-0.0073	-	0.0169	0.0061	-	3.0287
S+RBD+S2-22	0.893	-0.0138	-	0.0196	-	0.2434	-5.3933
S+RBD+S1-5+S2-22	0.894	-0.0129	-	0.0190	0.0010	0.2217	-4.7672
S1+RBD	0.726	-	0.0154	0.0090	-	-	2.3138
S1+RBD+S1-5	0.828	-	0.0064	0.0094	0.0061	-	1.6875
S1+RBD+S2-22	0.884	-	0.0030	0.0086	-	0.2395	-7.1780
S1+RBD+S1-5+S2-22	0.887		0.0025	0.0087	0.0014	0.2093	-6.1235
S+S1+RBD	0.750	-0.0252	0.0387	0.0195	-	-	4.0824
S+S1+RBD+S1-5	0.835	-0.0151	0.0209	0.0157	0.0058	-	2.7824
S+S1+RBD+S2-22	0.900	-0.0223	0.0238	0.0179	-	0.2361	-5.4763
S+S1+RBD+S1-5+S2-22	0.900	-0.0214	0.0228	0.0176	0.0006	0.2234	-5.0984

Supplementary Figure S6. Correlations between neutralization titers (authentic virus) and the combinations of SARS-CoV-2 proteins and peptides.

a Correlation of the NT50 (authentic virus) with combinations. **b** Linear regression equations for combinations.

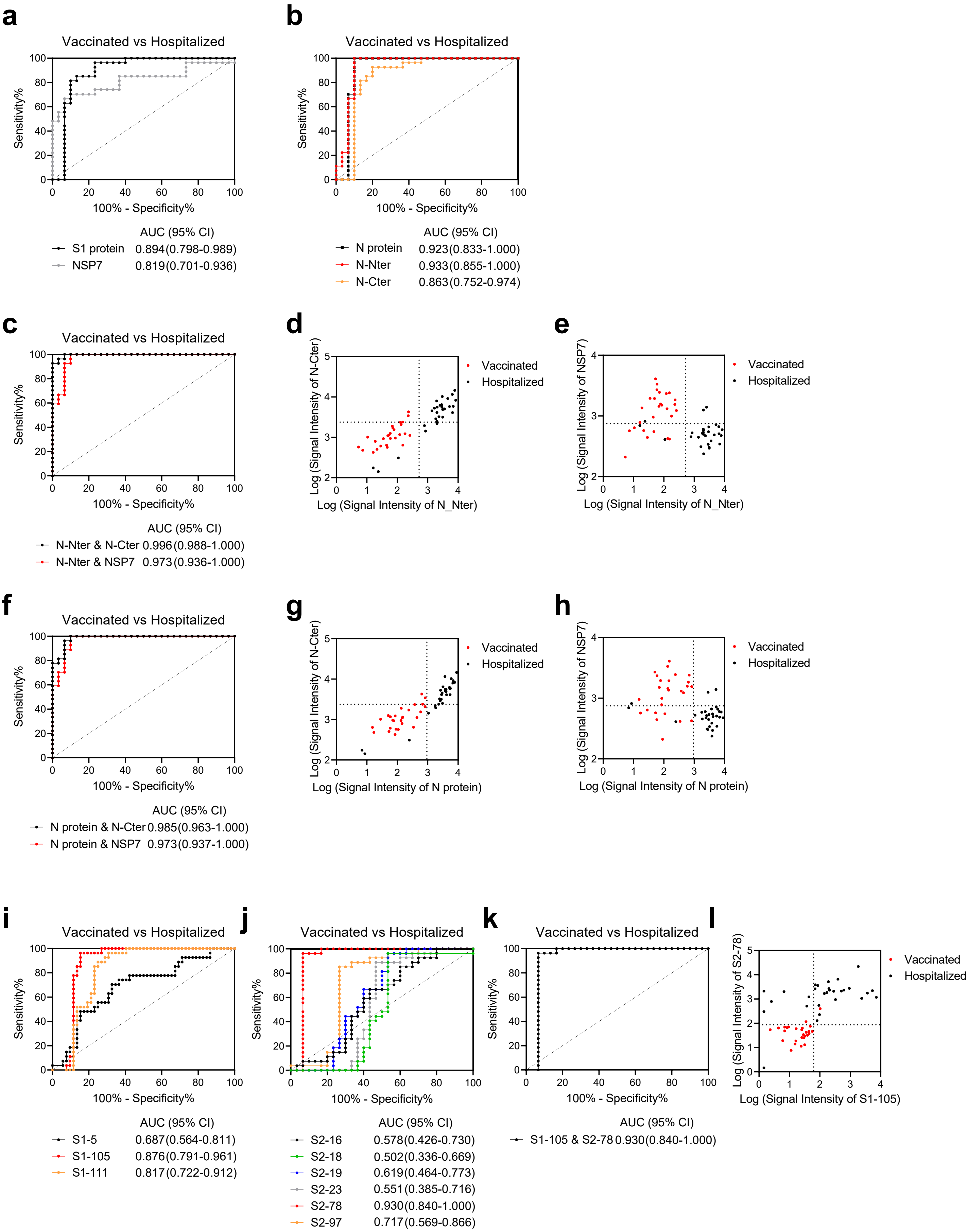
a
Vaccinated vs Hospitalized
Sensitivity%
100% - Specificity%
AUC (95% CI)
S1 protein 0.894(0.798-0.989)
NSP7 0.819(0.701-0.936)
b
Vaccinated vs Hospitalized
Sensitivity%
100% - Specificity%
AUC (95% CI)
N protein 0.923(0.833-1.000)
N-Nter 0.933(0.855-1.000)
N-Cter 0.863(0.752-0.974)
c
Vaccinated vs Hospitalized
Sensitivity%
100% - Specificity%
AUC (95% CI)
N-Nter & N-Cter 0.996(0.988-1.000)
N-Nter & NSP7 0.973(0.936-1.000)
d
Log (Signal Intensity of N-Cter)
Log (Signal Intensity of N_Nter)
Vaccinated
Hospitalized
e
Log (Signal Intensity of NSP7)
Log (Signal Intensity of N_Nter)
Vaccinated
Hospitalized
f
Vaccinated vs Hospitalized
Sensitivity%
100% - Specificity%
AUC (95% CI)
N protein & N-Cter 0.985(0.963-1.000)
N protein & NSP7 0.973(0.937-1.000)
g
Log (Signal Intensity of N-Cter)
Log (Signal Intensity of N protein)
Vaccinated
Hospitalized
h
Log (Signal Intensity of NSP7)
Log (Signal Intensity of N protein)
Vaccinated
Hospitalized
i
Vaccinated vs Hospitalized
Sensitivity%
100% - Specificity%
AUC (95% CI)
S1-5 0.687(0.564-0.811)
S1-105 0.876(0.791-0.961)
S1-111 0.817(0.722-0.912)
j
Vaccinated vs Hospitalized
Sensitivity%
100% - Specificity%
AUC (95% CI)
S2-16 0.578(0.426-0.730)
S2-18 0.502(0.336-0.669)
S2-19 0.619(0.464-0.773)
S2-23 0.551(0.385-0.716)
S2-78 0.930(0.840-1.000)
S2-97 0.717(0.569-0.866)
k
Vaccinated vs Hospitalized
Sensitivity%
100% - Specificity%
AUC (95% CI)
S1-105 & S2-78 0.930(0.840-1.000)
l
Log (Signal Intensity of S2-78)
Log (Signal Intensity of S1-105)
Vaccinated
Hospitalized

Supplementary Figure S7. Representative proteins and peptides for differentiating between vaccinated volunteers and hospitalized patients.

a-c ROC analysis of IgG responses to S1 (black) and NSP7 (gray) (**a**), the N protein (black), N-Nter (red) and N-Cter (orange) (**b**), and the combinations of N-Nter and N-Cter (black) or N-Nter and NSP7 (red) (**c**). **d-e** Scatter plots of IgG response of vaccinated volunteers (red dots) and convalescent patients (black dots) for N-Nter vs N-Cter (**d**) and N-Nter vs NSP7 (**e**). **f** ROC analysis of IgG responses to the combination of the N protein and N-Cter (black) and the N protein and NSP7 (red). **g-h** Scatter plots of IgG responses of vaccinated volunteers (red dots) and convalescent patients (black dots) for the N protein vs N-Cter (**g**) and the N protein vs NSP7 (**h**). **i-k** ROC analysis of IgG responses to S1-5 (black), S1-105 (red) and S1-111 (orange) (**i**), S2-16 (black), S2-18 (green), S2-19 (blue), S2-23 (gray), S2-78 (red) and S2-97 (orange) (**j**), and the combination of S1-105 and S2-78 (**k**). **l** Scatter plots of IgG response of vaccinated volunteers (red dots) and convalescent patients (black dots) for S1-105 vs S2-78. The gray lines in **d**, **e**, **g**, **h** and **l** indicate cutoff values based on the optimal Youden index of the related ROC curve. Unless otherwise stated, sera collected after the 2nd vaccine dose were analyzed.

Supplementary Table S1. Neutralization titer of the pseudovirus and the level of anti-RBD antibodies (TAb, IgG, IgM).

NT50 of pseudovirus				
Volunteer ID	1st dose 14 d	1st dose 28 d	2nd dose 21 d	2nd dose 28 d
#1	<30	/	/	/
#2	64.058	154.542	282.268	182.138
#3	<30	/	/	/
#4	429.732	/	/	/
#5	94.320	<30	/	/
#6	142.371	/	/	/
#7	53.786	/	/	/
#8	48.427	/	297.607	156.797
#9	334.847	79.090	191.583	/
#10	365.810	/	222.738	/
#11	186.517	176.167	236.344	151.175
#12	205.253	233.685	298.875	294.913
#13	117.799	188.523	/	/
#14	171.227	167.908	962.638	568.612
#15	100.887	/	247.312	/
#16	416.785	179.503	315.399	380.591
#17	127.585	/	156.355	208.266
#18	255.209	/	/	/
#19	/	239.770	152.520	/
#20	/	224.392	340.626	/
#21	/	<30	/	/
#22	/	105.075	/	/
#23	/	<30	/	/
#24	/	<30	/	/
#25	/	/	338.575	/
#26	/	/	624.118	/
#27	/	/	447.249	/
#28	/	/	508.227	/
#29	/	/	362.834	/
#30	/	/	296.064	/
#31	/	/	278.789	/
#32	/	/	/	209.676

Supplementary Table S1. Neutralization titer of the pseudovirus and the level of anti-RBD antibodies (TAb, IgG, IgM). (Continued)

	anti-RBD antibodies level						
Serum ID	A_{450}	A_{450}	A_{450} (15x dilution)	A_{450} (45x dilution)	A_{450} (135x dilution)	S/CO	S/CO (15x dilution)
#1-1	0.000	0.021	/	/	/	0.350	/
#2-1	0.214	0.029	/	/	/	0.620	/
#3-1	0.000	0.025	/	/	/	0.060	/
#4-1	0.078	0.512	/	/	/	7.320	/
#5-1	0.062	0.240	/	/	/	4.180	/
#6-1	0.144	0.162	/	/	/	2.550	/
#7-1	-0.001	0.010	/	/	/	0.010	/
#8-1	-0.003	0.009	/	/	/	0.110	/
#9-1	0.008	0.008	/	/	/	0.040	/
#10-1	0.168	0.667	/	/	/	0.570	/
#11-1	0.022	0.034	/	/	/	0.030	/
#12-1	0.107	0.778	/	/	/	5.410	/
#13-1	0.364	0.336	/	/	/	1.050	/
#14-1	0.027	0.169	/	/	/	0.100	/
#15-1	0.015	0.008	/	/	/	1.120	/
#16-1	0.007	0.056	/	/	/	0.050	/
#17-1	0.026	0.187	/	/	/	0.890	/
#18-1	0.042	0.063	/	/	/	0.330	/
#19-2	-0.004	0.085	/	/	/	0.030	/
#20-2	0.028	0.635	/	/	/	7.300	/
#21-2	0.681	1.612	/	/	/	180.090	/
#22-2	0.005	0.334	/	/	/	0.220	/
#23-2	0.024	0.011	/	/	/	0.050	/
#24-2	0.003	0.328	/	/	/	6.870	/
#25-3	0.169	2.706	0.287	0.095	0.041	20.930	/
#26-3	0.068	2.621	0.321	0.098	0.042	6.170	/
#27-3	0.002	2.109	0.123	0.044	0.027	0.190	/
#28-3	0.045	1.886	/	/	/	6.770	/
#29-3	0.010	1.677	/	/	/	2.690	/
#30-3	0.008	0.974	/	/	/	7.030	/
#31-3	0.092	3.048	1.290	0.439	0.157	194.150	/
#32-4	0.025	2.356	0.277	0.097	0.041	18.910	/
#2-2	0.005	0.021	/	/	/	0.040	/
#5-2	0.059	0.450	/	/	/	13.850	/
#8-3	0.014	1.991	0.136	0.039	0.020	3.500	/
#9-2	0.015	0.095	/	/	/	0.080	/
#10-3	0.360	2.587	0.226	0.076	0.031	12.220	/
#11-2	0.022	0.128	/	/	/	1.030	/
#12-2	0.041	1.224	/	/	/	0.830	/
#13-2	0.100	0.362	/	/	/	0.220	/
#14-2	0.147	0.324	/	/	/	0.190	/
#15-3	0.708	2.999	0.546	0.165	0.051	32.340	/
#16-2	0.231	0.150	/	/	/	4.150	/
#17-3	0.174	2.275	0.309	0.108	0.041	2.760	/
#19-3	0.010	0.791	/	/	/	0.360	/
#20-3	0.093	1.676	/	/	/	19.390	/
#2-3	0.222	1.376	/	/	/	12.150	/
#8-4	-0.001	1.776	/	/	/	12.240	/
#9-3	0.002	1.969	0.131	0.038	0.015	5.580	/
#11-3	0.585	2.247	0.204	0.077	0.037	36.480	/
#12-3	0.063	3.112	0.473	0.160	0.047	15.340	/
#14-3	2.139	3.073	0.619	0.209	0.073	154.680	/
#16-3	0.058	2.325	0.216	0.062	0.026	7.100	/
#17-4	0.185	2.934	0.509	0.171	0.065	66.570	/
#2-4	0.256	1.524	/	/	/	17.000	/
#11-4	0.264	2.297	0.226	0.094	0.035	32.330	/
#12-4	0.066	2.917	0.408	0.130	0.039	4.890	/
#14-4	1.599	3.112	0.527	0.178	0.060	155.880	/
#16-4	0.013	2.334	0.215	0.066	0.019	/	3.490

Supplementary Table S2. Neutralization titers of authentic SARS-CoV-2.

	NT50 of authentic virus			
Volunteer ID	1st dose 14 d	1st dose 28 d	2nd dose 21 d	2nd dose 28 d
#1	9.075	/	/	/
#2	6.221	10.940	20.540	14.760
#3	0.000	/	/	/
#4	ND	/	/	/
#5	13.760	ND	/	/
#6	ND	/	/	/
#7	0.000	/	/	/
#8	ND	/	19.710	15.340
#9	0.000	0.000	3.628	/
#10	ND	/	23.080	/
#11	5.402	7.402	10.110	2.196
#12	14.830	5.178	18.790	19.040
#13	11.270	20.390	/	/
#14	3.837	14.040	171.400	113.200
#15	0.000	/	ND	/
#16	2.826	0.000	11.910	ND
#17	ND	/	24.700	ND
#18	14.660	/	/	/
#19	/	0.000	5.190	/
#20	/	0.000	46.470	/
#21	/	ND	/	/
#22	/	ND	/	/
#23	/	ND	/	/
#24	/	ND	/	/
#25	/	/	31.370	/
#26	/	/	58.680	/
#27	/	/	45.510	/
#28	/	/	13.570	/
#29	/	/	1.564	/
#30	/	/	12.250	/
#31	/	/	59.370	/
#32	/	/	/	ND
